# A Scallop Active Peptide-Incorporated Carboxymethyl Chitosan/Oxidized Sodium Alginate Hydrogel for Accelerating Skin Wound Healing and Tissue Regeneration

**DOI:** 10.3390/gels12060467

**Published:** 2026-05-28

**Authors:** Lingfeng Kou, Huan Yang, Yunquan Zheng

**Affiliations:** 1 College of Chemistry, Fuzhou University, No. 2 Xueyuan Road, Fuzhou 350108, China; 17346034872@163.com (L.K.); 241320331@fzu.edu.cn (H.Y.); 2 Fujian Key Laboratory of Medical Instrument and Pharmaceutical Technology, Fuzhou University, No. 2 Xueyuan Road, Fuzhou 350108, China

**Keywords:** scallop active peptide, angiogenesis, antioxidant, hydrogel, wound healing

## Abstract

In this study, a novel composite hydrogel was developed based on oxidized sodium alginate (OSA), synthesized via sodium periodate oxidation, and incorporated into a carboxymethyl chitosan (CMCS) matrix. Scallop active peptides (SAPs), a marine-derived bioactive component with outstanding antioxidant and pro-regenerative activities, was introduced to endow the hydrogel with enhanced biological functions, which is of great significance for breaking the functional limitations of traditional single-component hydrogels. The construction of a dynamic covalent network, driven by the Schiff base reaction, was confirmed through structural characterization using FT-IR and ^1^H-NMR. The hydrogel exhibited favorable physicochemical properties, including shear-thinning behavior, significant self-healing capability, and a uniform porous microstructure that effectively mimics the extracellular matrix (ECM). In vitro evaluations revealed excellent biocompatibility and potent pro-angiogenic potential, as evidenced by enhanced HUVEC migration and tube formation. In a rat model of full-thickness skin wounds, the CMCS/OSA/SAPs hydrogel significantly accelerated wound closure and promoted re-epithelialization and organized collagen deposition. Furthermore, immunohistochemical analysis confirmed upregulated VEGF and α-SMA expression, alongside reduced inflammatory levels (decreased iNOS), indicating potent tissue-regenerative and immunomodulatory functions. Overall, this work presents a multifunctional hydrogel system that integrates antioxidant, anti-inflammatory, and tissue-regenerative properties, offering a promising strategy for deep-wound healing. This study highlights the significant potential of marine-derived bioactive proteins/peptides in the development of advanced biomedical materials.

## 1. Introduction

As the largest organ of the human body, the skin integrates several key physiological functions, including biological barrier protection, moisture maintenance, and physical perception [1]. Serving as the physical interface between the body and the external environment, it constitutes the primary line of defense against the invasion of pathogenic microorganisms and excessive water loss [2]. However, severe trauma, burns, surgical procedures, and chronic diseases can compromise skin integrity, leading to intense inflammatory responses and wound infections [3]. Skin repair is a complex and dynamic process consisting of four overlapping stages: hemostasis, inflammation, proliferation, and tissue remodeling [4]. Currently, the clinical treatment of large-area or chronic non-healing wounds remains highly dependent on autologous skin grafting; however, this method is constrained by limited donor sources, the risk of secondary trauma, and the potential for hypertrophic scarring [5,6]. Consequently, the development of synthetic substitute materials with excellent biocompatibility has become a focal point of research in the field of tissue engineering.

Traditional dressings, such as gauze and bandages, are cost-effective and highly absorbent. However, they tend to adhere to the wound surface, leading to secondary trauma upon removal, and the dry environment they create can inhibit keratinocyte migration and the activity of growth factors [7,8]. Modern medical research has confirmed that a moist environment can significantly accelerate the rate of wound healing [9]. Against this background, hydrogel dressings have emerged as a superior alternative due to their high water content and biomimetic structure, which closely simulate the extracellular matrix (ECM) in terms of physicochemical properties, thereby demonstrating excellent biocompatibility and low immunogenicity [10]. During wound repair, hydrogels not only maintain a moist healing environment and promote cell proliferation but also serve as an effective physical barrier to block bacterial invasion [11,12]. Furthermore, the physical cooling effect of hydrogels can alleviate pain, and their non-adherent nature prevents damage to newly formed tissues. These properties have led to significant clinical advantages in the treatment of diabetic foot ulcers and burns [13]. However, traditional natural polymer hydrogels also possess inherent limitations, such as limited mechanical strength and vulnerability to rapid dehydration [14]. Furthermore, common sterilization protocols can alter polymer networks and compromise structural integrity, affecting chemical structure, swelling behavior and mechanical properties [15]. Therefore, developing composite hydrogels that combine enhanced mechanical performance with structural stability under mild or compatible sterilization conditions remains a critical challenge for biomedical translation [16].

Among various hydrogel systems, Carboxymethyl chitosan (CMCS) has garnered significant attention due to its excellent water solubility and ability to promote fibroblast migration. Meanwhile, oxidized sodium alginate (OSA), enriched with reactive aldehyde groups, can undergo a spontaneous Schiff base reaction with the amino groups of CMCS to form a hydrogel with a dynamic cross-linked network [17,18]. This system not only mimics the physical properties of the ECM but also exhibits remarkable self-healing capabilities and biodegradability, allowing it to adapt flexibly to wound areas with complex shapes [19]. Nevertheless, while CMCS offers excellent biocompatibility, its standalone gelation is structurally weak [20]; conversely, OSA provides rich reactive aldehyde groups for rapid Schiff base cross-linking but lacks cell-adhesive sites [21]. Crucially, although both polymers form a supportive structural matrix, they fundamentally lack the intrinsic biological activity required to actively guide cell proliferation and tissue regeneration [22,23]. To bridge these physicochemical and biological gaps, the integration of an active substance into this dynamic network is indispensable [24]. However, for full-thickness skin defects, the restoration of tissue integrity is often hindered by a pathological microenvironment characterized by severe oxidative stress [25,26]. While traditional pure hydrogel matrices can provide physical protection, they often lack the biological activity necessary to actively modulate such complex biochemical processes, remaining insufficient in addressing intense inflammatory responses and multiple pathological damages [27,28].

Oxidative stress resulting from reactive oxygen species (ROS) overproduction is a central hallmark of impaired wound healing [29]. Excessive ROS can trigger cellular apoptosis, prolong the inflammatory phase, and hinder the transition of the wound from the inflammatory stage to the proliferative stage [30]. Consequently, integrating active factors with multiple biological functions into hydrogels has become a significant research focus. Marine-derived bioactive substances, particularly scallop active peptides (SAPs), have demonstrated unique potential for biomedical applications [31]. Research by Zhi, T. and Wang, Z. [32,33] revealed that SAPs extracted from fresh scallop adductor muscle possess significant antioxidant properties, effectively neutralizing free radicals and mitigating oxidative cellular damage. Furthermore, the anti-photoaging activity reported by Liu, X. [34] suggests the profound potential of SAPs in maintaining skin cell homeostasis and promoting tissue remodeling. We hypothesize that the incorporation of SAPs not only creates a healing environment with low oxidative stress but also constructs a biological barrier against infection and oxidative damage through the synergistic effects of its multiple activities. This approach is expected to provide multi-dimensional support for the rapid repair of deep-seated wounds.

In brief, the selection of these ingredients is based on a complementary design: CMCS and OSA establish a dynamic physical skeleton via spontaneous Schiff base cross-linking to maintain structural integrity and a sufficient safety margin for skin tissue adaptation, while the interpolated SAPs enrich the matrix with vital bioactivities to mitigate oxidative stress and promote tissue repair. This rational integration effectively bridges the drawbacks of individual components, providing a balanced biological microenvironment for promoting wound healing.

Based on these considerations, this study aims to address critical clinical challenges in full-thickness skin defect care—specifically, the pathological microenvironment driven by severe oxidative stress and inadequate vascularization that typically stalls regeneration. To resolve these limitations, we developed a novel multifunctional composite bioactive hydrogel CMCS/OSA/SAPs. By leveraging the bioactivities of SAPs, this formulation is designed to scavenge excessive ROS and accelerated tissue repair. We systematically characterized the physicochemical properties, rheological stability, and in vitro antioxidant activity of the composite hydrogel. Furthermore, a Sprague-Dawley (SD) rat full-thickness skin defect model was utilized to verify its in vivo therapeutic efficacy. Through histological evaluations—including H&E and Masson’s trichrome staining, as well as CD31 and α-SMA immunohistochemical analysis, we focused on investigating the system’s comprehensive performance in accelerating re-epithelialization, inhibiting wound inflammation, promoting collagen deposition, and inducing the maturation of nascent blood vessels. This research provides a new strategy for the development of high-performance wound dressings utilizing marine-derived bioactive materials.

## 2. Results and Discussion

### 2.1. Preparation and Characterization of OSA

**Figure 1:**
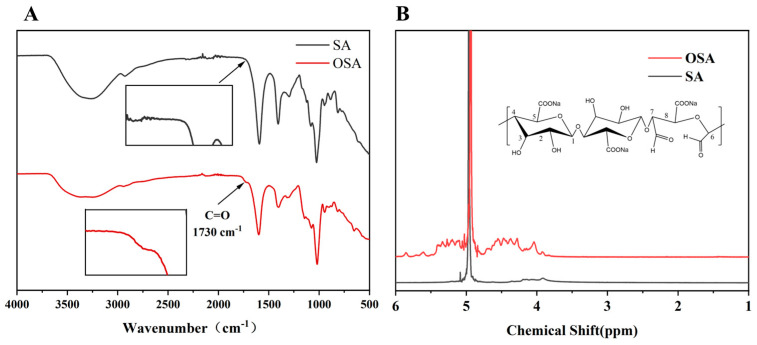
Preparation and characterization of OSA. (**A**) FT-IR of SA and OSA; (**B**) ^1^H-NMR of SA and OSA.

To verify the successful synthesis of OSA from SA via NaIO4-mediated oxidation, a comparative analysis was performed using Fourier-transform infrared (FT-IR) and ^1^H nuclear magnetic resonance (^1^H-NMR) spectroscopies. As shown in the FT-IR spectra (Figure 1A), the characteristic profiles of SA and OSA are generally similar within the wavenumber range of 4000–400 cm^−1^. However, a distinct new absorption peak emerged at 1730 cm^−1^ in the OSA spectrum, which is attributed to the symmetric stretching vibration of the aldehyde carbonyl groups generated during oxidation.

The ^1^H NMR spectra further substantiated the structural transition (Figure 1B). The signal at *δ* = 4.79 ppm corresponds to the residual proton resonance of the solvent (D_2_O). For both samples, the overlapping multiplets in the range of *δ* = 3.50–4.50 ppm are assigned to the H2–H5 protons of the uronic acid units. In the OSA spectrum, the emergence of new signals between δ = 4.30 and 5.40 ppm is associated with the H6 and H7 protons of the oxidized uronic acid residues. Notably, the characteristic peaks at *δ* = 5.61 and 5.84 ppm are attributed to the hemiacetal protons formed by the reaction between the newly generated aldehyde groups and adjacent hydroxyl groups. Collectively, the FT-IR and ^1^H NMR results confirm the successful preparation of OSA with the expected functional groups.

### 2.2. Preparation and Characterization of Hydrogels

**Figure 2:**
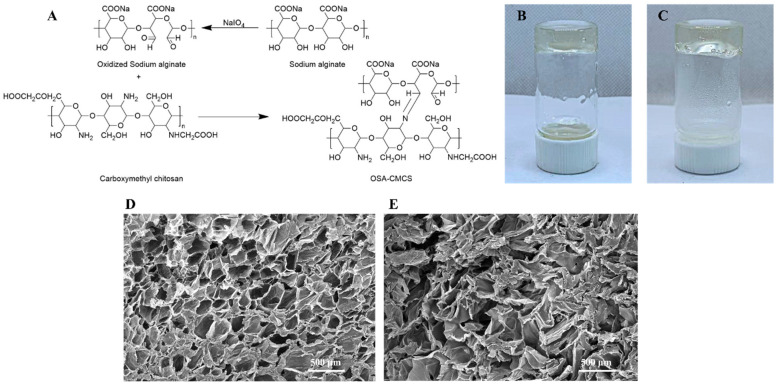
Preparation and morphological characterization of hydrogels. (**A**) Flow chart of the preparation of CMCS/OSA hydrogels; (**B**) macroscopic morphology of CMCS/OSA hydrogel; (**C**) macroscopic morphology of CMCS/OSA/SAPs hydrogel; (**D**) SEM images of CMCS/OSA; (**E**) SEM images of CMCS/OSA/SAPs.

Figure 2A illustrates the comprehensive fabrication process for the CMCS/OSA hydrogels. Regarding the CMCS/OSA/SAPs composite hydrogels, SAPs was encapsulated by direct addition into the precursor solution before cross-linking.

#### 2.2.1. Macroscopic Morphological Analysis

The macroscopic appearances of the CMCS/OSA and CMCS/OSA/SAPs hydrogels are presented in Figure 2B and Figure 2C, respectively. Both hydrogels exhibited excellent macroscopic homogeneity immediately following fabrication, with no apparent macromolecular aggregation or phase separation. The CMCS/OSA hydrogel was characterized by a colorless and transparent appearance, displaying a uniform, bubble-free network. While the CMCS/OSA/SAPs composite hydrogel remained colorless and transparent, a minor presence of microbubbles was observed. This phenomenon is likely attributed to the surface-active properties inherent in the amphiphilic structure of SAPs; these microbubbles did not compromise the structural integrity of the hydrogels, ensuring their suitability for subsequent performance evaluations and bioactivity assays.

#### 2.2.2. Microscopic Morphological Analysis

The micromorphology of the hydrogels was characterized via SEM. As illustrated in Figure 2D, the CMCS/OSA hydrogel, cross-linked through dynamic covalent bonds, exhibited a highly interconnected and uniformly distributed porous three-dimensional 3D network structure. Upon the incorporation of SAPs (Figure 2E), the CMCS/OSA/SAPs composite hydrogel retained its superior porous morphology; although a slight variation in pore size was observed, the overall structural connectivity remained unimpaired. This highly biomimetic architecture effectively recapitulates the microenvironment of the ECM, providing an ideal scaffold for cell adhesion, migration, and proliferation. Furthermore, the high specific surface area inherent in this porous structure endows the material with excellent liquid absorption and water retention capacities, enabling it to effectively manage excess wound exudate and subsequently promote the wound healing process.

#### 2.2.3. Rheological Results

**Figure 3:**
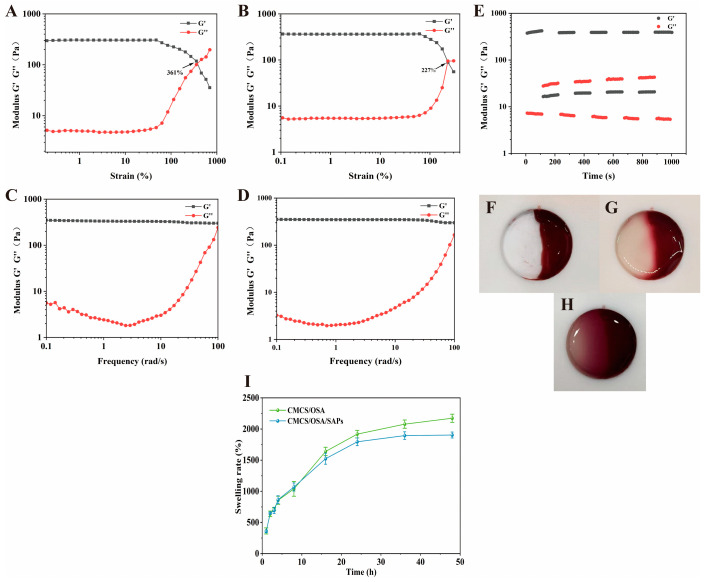
The rheological properties and swelling performance of hydrogels. (**A**) Strain sweep of CMCS/OSA; (**B**) strain sweep of CMCS/OSA/SAPs; (**C**) frequency sweep of CMCS/OSA; (**D**) frequency sweep of CMCS/OSA/SAPs; (**E**) step-strain test of CMCS/OSA/SAPs; (**F**–**H**) 0, 2, 24 h macroscopic image of self-healing CMCS/OSA/SAPs hydrogel; (**I**) the swelling curves of different hydrogels.

The structural integrity and dynamic properties of the hydrogels were systematically evaluated via rheological characterization. As illustrated in the amplitude sweep profiles presented in Figure 3A,B, both the CMCS/OSA and CMCS/OSA/SAPs hydrogels exhibited a broad linear viscoelastic region (LVR). At low strain amplitudes, the storage modulus G′ consistently exceeded the loss modulus G″, confirming the formation of a stable and elastic 3D network. As the strain surpassed the critical threshold, a crossover of G′ and G″ occurred—at 361% for the CMCS/OSA group and 227% for the SAPs-incorporated group—signifying the structural collapse of the hydrogel matrix under large-scale deformation. Notably, the reduction in the critical strain threshold upon the integration of SAPs is primarily attributed to the steric hindrance effects introduced by the exogenous peptide chains [35]. The interpolation of SAPs into the polysaccharide network partially disturbs the continuous alignment of CMCS and OSA macromolecules, restricting their chain mobility and sliding capacity under external shear, which subsequently leads to a premature breakdown of the dynamic cross-linked framework [36,37]. Nevertheless, it is worth emphasizes that a critical strain threshold of 227% remains highly sufficient to support the practical requirements of skin-targeted wound dressings. Given that natural human skin rarely experiences localized deformations exceeding 50–100% [38] during physiological movements, the developed CMCS/OSA/SAPs hydrogel offers an ample structural safety margin, ensuring that the dressing maintains its mechanical integrity and continuous physical barrier on dynamic wound sites without premature structural fragmentation [39].

The frequency sweep results depicted in Figure 3C,D further demonstrated that the hydrogels exhibited frequency-independent behavior over the range of 0.1 to 100 rad/s. Throughout the tested frequency spectrum, G′ maintained its predominance over G″, highlighting the exceptional stability and robust cross-linked network of the composite systems.

The self-healing efficiency of the hydrogels was quantitatively assessed via continuous step-strain measurements, as presented in Figure 3E. When the applied strain was periodically cycled between a large-amplitude oscillatory strain and a low strain, the G′ and G″ values recovered to their initial states almost instantaneously. This rapid recovery is primarily attributed to the reversible dissociation and autonomous reformation of dynamic Schiff base bonds formed between the amino groups of CMCS and the aldehyde groups of OSA. The macroscopic observations depicted in Figure 3F–H are consistent with these rheological findings: two separate hydrogel fragments gradually merged into a monolithic and seamless entity over time, with the interface disappearing completely. Collectively, these results indicate that the incorporation of SAPs does not impair the inherent self-healing capacity or structural stability of the hydrogel, making it an ideal candidate for dynamic wound management applications.

From a clinical application perspective that the hydrogels possessed stable viscoelastic properties and structural integrity. The LVR profiles demonstrate a stable G′ dominant over the G″, imparting an appropriate structural firmness to maintain physical coverage on open wounds. Concurrently, the distinct gel-to-sol transition observed under elevated strain suggests that the hydrogel possesses efficient shear-responsive manipulability. Under practical clinical delivery, the hydrogel network can readily adjust its viscoelastic state to ensure excellent spreadability over irregular wound beds. Furthermore, the rapid, complete recovery of G′ under cyclic strain, combined with the macroscopically observed self-healing capability, provides a strong adhesive safeguard to adapt to frequent tissue motion without detachment, the inversion test further confirmed the self-supporting nature and shape-retention capability of the hydrogels, indicating that the materials could remain stable without obvious flow under gravity, such characteristics may contribute to favorable handling performance and adaptability during topical wound application.

#### 2.2.4. Analysis of Swelling

The swelling kinetics of the CMCS/OSA and CMCS/OSA/SAPs hydrogels are illustrated in Figure 3I. The swelling ratios were evaluated over a 48 h period, during which both hydrogel formulations exhibited comparable swelling profiles. During the initial 10 h, a burst swelling phase was observed in both groups, characterized by extremely rapid water uptake. This phenomenon is typically attributed to the rapid diffusion of water molecules into the 3D polymer network and the instantaneous hydration of hydrophilic moieties. As the experiment progressed, the swelling rate gradually decreased and eventually reached a plateau, indicating that the internal osmotic pressure within the hydrogel matrix had achieved equilibrium with the elastic restorative force of the cross-linked network.

Specifically, the blank CMCS/OSA hydrogel exhibited a higher swelling capacity, reaching a maximum swelling ratio of approximately 2173.46% at 48 h, and maintained a subtle upward trend even in the terminal stages. In contrast, the incorporation of SAPs led to a moderate reduction in swelling capacity, with the CMCS/OSA/SAPs hydrogel stabilizing at a swelling ratio of approximately 1903.58%. Although the introduction of SAPs exerted a discernible influence on the swelling efficiency, the values remained within the optimal range for bioactive dressings, providing sufficient capacity to maintain a moist microenvironment at the wound site without compromising structural stability. Similar swelling behavior has also been reported in other double-network polysaccharide hydrogels based on CMCS/OSA or CS/SA derivative systems [18,40], where the incorporation of bioactive components slightly reduced the swelling ratio while still maintaining sufficient water absorption capacity and structural stability. This phenomenon may be attributed to the enhanced intermolecular interactions within the hydrogel network, resulting in a denser internal structure and partially restricted water diffusion. Morseover, the high swelling capacity enables the hydrogels to effectively absorb excessive wound exudate, which may help prevent fluid accumulation and provide a favorable environment for wound healing. Collectively, these findings suggest that this structural modulation does not interfere with the material’s functional performance, and the integrated SAPs confers enhanced biofunctionality, reinforcing the potential of CMCS/OSA/SAPs hydrogels as advanced functional wound dressings.

### 2.3. Biocompatibility and Hemocompatibility

**Figure 4:**
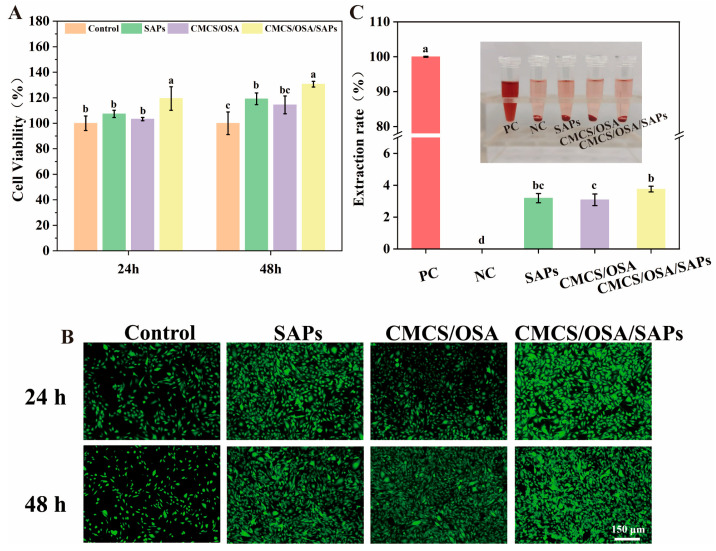
Biocompatibility and hemocompatibility. (**A**) Cell viability of HUVEC cells after 24 and 48 h of incubation with different samples measured by CCK-8 assay; (**B**) fluorescence images of live/dead stained cells after 24 and 48 h; (**C**) hemolysis ratio of different samples (PC: positive control, NC: negative control). Data are presented as mean ± SD (*n* = 3). Different letters (a–d) indicate significant differences (*p* < 0.05).

The cytocompatibility of both the CMCS/OSA and CMCS/OSA/SAPs hydrogel extracts was systematically evaluated, demonstrating that all formulations supported the growth and proliferation of HUVECs. After 24 h of co-culture, the cell viability in all experimental groups exceeded that of the control group, with the CMCS/OSA/SAPs composite hydrogel group exhibiting the highest viability at 119.43 ± 9.21%. Following 48 h of co-culture, the proliferation-promoting effects became more pronounced compared to the 24 h interval. The SAPs-incorporated composite hydrogel continued to demonstrate superior cell viability at 130.64 ± 2.25%, effectively inducing in vitro cellular proliferation. The live/dead staining results for HUVECs after 24 h and 48 h of co-culture are illustrated in Figure 4B. After the initial 24 h period, abundant green fluorescence was observed across all experimental groups, with the SAPs-incorporated composite hydrogel group displaying the highest cell density and fluorescence distribution. By 48 h, both fluorescence intensity and cell density had significantly increased across all groups compared to the 24 h observations. Notably, the CMCS/OSA/SAPs group maintained the most robust green fluorescence intensity. These live/dead staining findings were in good agreement with the CCK-8 results, collectively confirming that all hydrogel formulations possess excellent cytocompatibility, with the SAPs-incorporated group significantly enhancing the in vitro proliferation of HUVECs.

During the wound healing process, dressings are inevitably in direct contact with wound exudate and blood; thus, superior hemocompatibility is a prerequisite for ensuring their biosafety. The hemocompatibility of the hydrogel dressings and their active components was quantitatively assessed using a hemolysis assay. As illustrated in Figure 4C, the supernatants from the SAPs, CMCS/OSA, and CMCS/OSA/SAPs composite hydrogel groups exhibited a clarified appearance comparable to that of the negative control (saline solution). In stark contrast, the positive control (Triton X-100) group displayed a distinct bright red color, signifying the complete lysis of erythrocytes under the action of the potent surfactant. Calculated results indicated that the hemolysis rates for all experimental groups were well below the international safety threshold of 5%. These findings further substantiate that both the CMCS/OSA hydrogel and the SAPs-incorporated composite hydrogel do not induce erythrocyte damage. Consequently, these materials possess excellent hemocompatibility and fulfill the stringent requirements for medical-grade biomaterials in clinical wound management.

### 2.4. In Vitro Biological Evaluation

#### 2.4.1. Cell Migration Results

**Figure 5:**
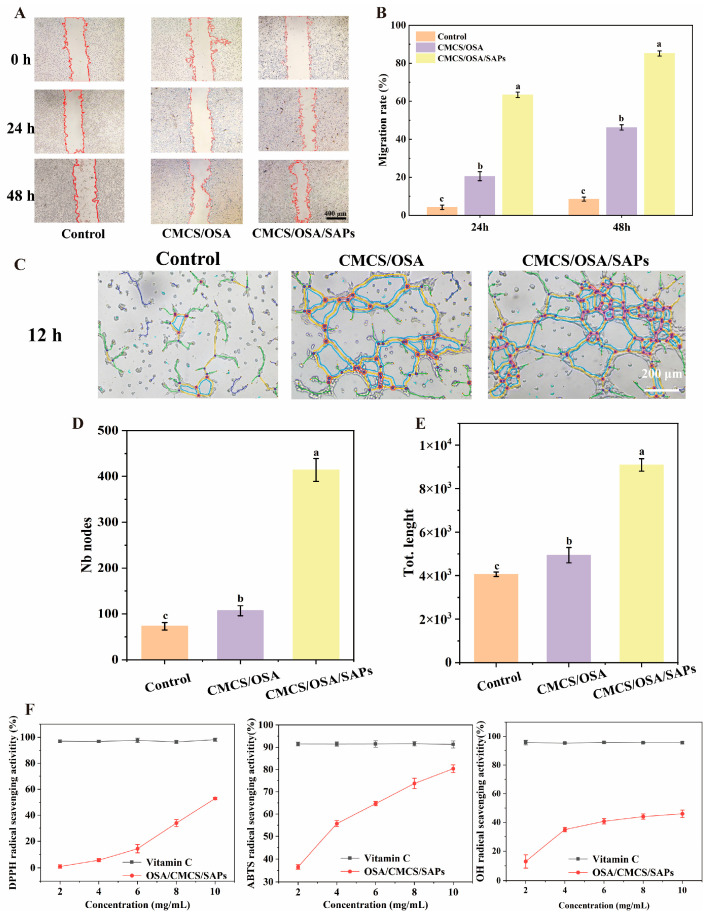
Evaluation of cell migration, angiogenesis, and antioxidant activity. (**A**) Representative images of the scratch wound healing assay after 0, 24, and 48 h of treatment; (**B**) quantitative analysis of the cell migration rate derived from the scratch assay; (**C**) optical images of the tube formation assay using HUVECs after 12 h of incubation; (**D**,**E**) statistical analysis of (**D**) the number of nodes (Nb nodes) and (**E**) the total tube length (Tot. length) in the tube formation assay; (**F**) antioxidant activities of the samples: DPPH^+^·, ABTS^+^·,and ·OH radical scavenging activities as a function of concentration. Data are presented as mean ± SD (*n* = 3). Different letters (a–c) indicate significant differences (*p* < 0.05).

As illustrated in the representative optical micrographs in Figure 5A, varying degrees of scratch closure were observed across all experimental groups over time. After 24 h of co-culture, the CMCS/OSA/SAPs composite hydrogel group exhibited the most significant centripetal migration and the highest cell density within the scratched zone compared to the other groups, indicating that the CMCS/OSA/SAPs system possesses the most potent pro-migratory activity. Following 48 h of co-culture, the denuded area in the CMCS/OSA/SAPs group achieved near-complete closure, whereas the other experimental groups still retained substantial blank areas. Quantitative analysis of the cell migration rates is presented in Figure 5B. The migration rates for the SAPs-incorporated composite hydrogel group reached 63.4 ± 1.44% and 85.2 ± 1.35% after 24 h and 48 h, respectively. Although the blank CMCS/OSA hydrogel also facilitated cell motility to some extent, its efficacy was significantly inferior to that of the composite system. These findings are in high alignment with the trends observed in the preceding cell proliferation assays. Collectively, these results demonstrate that the CMCS/OSA/SAPs composite hydrogel can effectively stimulate the migratory potential of endothelial cells, thereby playing a pivotal role in accelerating the wound healing process.

#### 2.4.2. Angiogenesis Results

As illustrated in Figure 5C, the control and CMCS/OSA hydrogel groups exhibited only sparse and fragmented tubular structures, failing to establish a cohesive vascular network. In contrast, the CMCS/OSA/SAPs composite hydrogel group demonstrated potent pro-angiogenic activity, characterized by the formation of robust and well-defined capillary-like networks composed of interconnected HUVECs. Quantitative analysis of the angiogenic parameters is presented in Figure 5D,E, focusing on the number of vascular nodes and the total tube length. The composite hydrogel group significantly outperformed the other experimental groups, yielding node counts and total lengths that were markedly higher than those of the control group. Consistent with the morphological observations, these quantitative findings substantiate the superior angiogenic capacity of the composite system.

This enhanced bioactivity may be attributed to the synergistic effect between the bioactive peptide SAPs and the CMCS/OSA hydrogel matrix. Specifically, the SAPs peptide facilitates the accelerated proliferation and directed migration of endothelial cells, while the biomimetic porous architecture of the CMCS/OSA hydrogel provides an ideal physical microenvironment for cellular infiltration and structural organization. Consequently, the CMCS/OSA/SAPs composite hydrogel demonstrates substantial potential for clinical applications aimed at promoting accelerated wound healing through enhanced neovascularization. Similar pro-angiogenic phenomena have also been reported [41] in marine-derived bioactive peptide-based biomaterials, where the regulation of oxidative stress and endothelial cell behavior played critical roles in vascular regeneration during wound healing. Notably, scallop-derived biomimetic materials have demonstrated the ability to accelerate tissue repair by promoting angiogenesis and modulating the wound microenvironment. These previous findings are consistent with the present results and further indicate that SAPs may possess intrinsic bioactive properties beneficial for endothelial activation and neovascularization.

#### 2.4.3. Antioxidant Activity

The in vitro antioxidant capacity of the composite hydrogels was evaluated by quantifying their scavenging efficiency against DPPH^+^·, ABTS^+^·, and ·OH radicals, as illustrated in Figure 5F. The scavenging activity of the composite hydrogels exhibited a clear dose-dependent relationship with the concentration of incorporated SAPs. Specifically, the radical scavenging rates increased significantly with elevated SAPs content, peaking at 56.42 ± 0.92%, 79.85 ± 1.73%, and 41.21 ± 2.53% for the respective radical species.

During the wound healing process, persistent pathological oxidative stress typically leads to the accumulation of excessive ROS within the wound microenvironment. This ROS overload can trigger the apoptosis of critical cellular components, such as endothelial cells and fibroblasts, thereby stalling the healing process. The CMCS/OSA/SAPs composite hydrogel effectively scavenges these excess ROS, thereby mitigating oxidative damage and fostering a favorable microenvironment for tissue regeneration.

The pronounced antioxidant activity of the CMCS/OSA/SAPs hydrogel is likely associated with the intrinsic bioactivity of marine-derived peptide fragments. Previous studies on shellfish-derived bioactive peptides have demonstrated that amino acid residues possessing electron-donating capacity [42], particularly hydrophobic and aromatic residues, contribute significantly to free radical scavenging activity. Therefore, the antioxidant performance observed in the present study may be partially attributed to the bioactive peptide components incorporated within the hydrogel network, which could help alleviate excessive ROS-induced oxidative damage. In addition, the CMCS/OSA hydrogel matrix may provide a favorable physicochemical microenvironment for maintaining peptide stability and facilitating interactions between the bioactive components and ROS molecules [43]. The synergistic effect between the antioxidant SAPs and the dynamic hydrogel network may collectively contribute to oxidative microenvironment regulation during wound healing.

### 2.5. Hydrogel’s Ability to Promote Wound Healing

**Figure 6:**
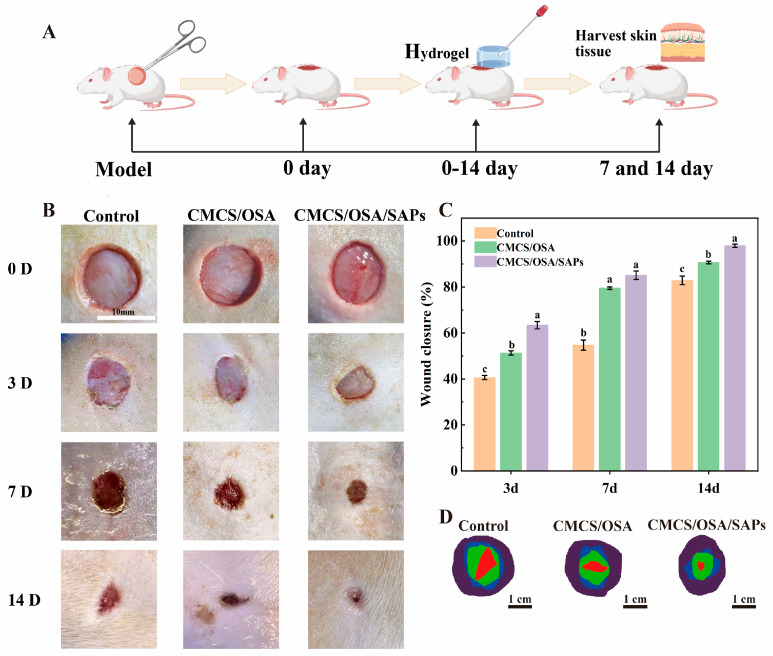
The wound healing of the CMCS/OSA/SAPs hydrogel. (**A**) Schematic illustration of the surgical procedure for creating the skin wound model and the subsequent treatment protocol over 14 days; (**B**) representative digital photographs of the wounds in the Control, CMCS/OSA, and CMCS/OSA/SAPs groups at 0, 3, 7, and 14 days post-surgery; (**C**) quantitative analysis of the wound closure rate at different time points (*n* = 3); (**D**) schematic diagrams (wound traces) showing the dynamic process of wound shrinkage for each group over 14 days. Data are expressed as mean ± SD. Different letters (a–c) indicate significant differences (*p* < 0.05).

The therapeutic efficacy of the CMCS/OSA/SAPs composite hydrogel in accelerating wound repair was evaluated using an SD rat full-thickness cutaneous wound model. The study included a blank control group and multiple experimental groups to systematically investigate the healing-promoting effects of the composite components on tissue regeneration. A simplified schematic of the overall experimental procedure is illustrated in Figure 6A.

Figure 6B shows images of the various stages of wound healing in each group. The quantitative analysis of the healing rate is shown in Figure 6C. Morphological assessment and quantitative analysis of the wound healing rates indicated that the CMCS/OSA/SAPs composite hydrogel significantly accelerated the re-epithelialization process. By day 3 of treatment, the wound healing rate in the composite hydrogel group reached 63.42%, which was significantly higher (*p* < 0.05) than the 42.18% observed in the blank control and other experimental groups, demonstrating superior potential for early-stage wound healing. Throughout the treatment period, the composite hydrogel group consistently maintained the most rapid contraction rate. By day 14, wounds treated with the composite hydrogel achieved near-complete closure with a healing rate of 97.95%, whereas varying degrees of residual defects remained observable in the other groups.

These in vivo findings substantiate that the CMCS/OSA/SAPs composite hydrogel not only provides effective physical protection for the wound site but also significantly enhances the overall healing kinetics.

### 2.6. Histological Analysis of Regenerated Wound Tissues

#### 2.6.1. Hematoxylin and Eosin Staining

**Figure 7:**
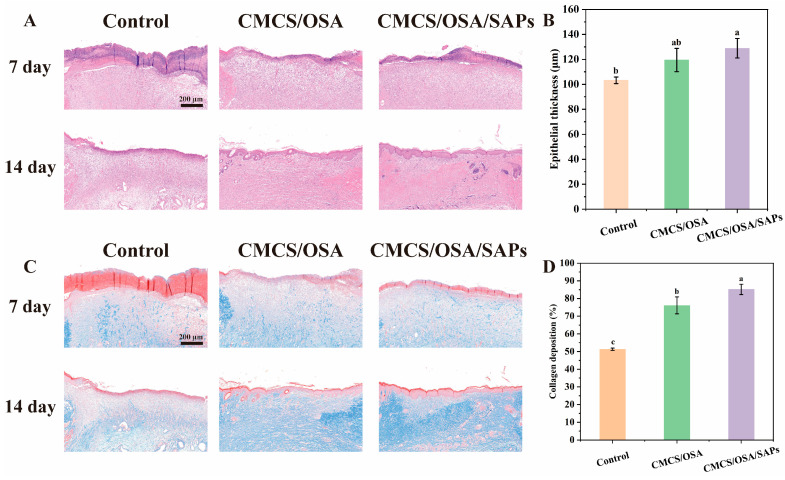
Histological analysis of the regenerated skin tissues. (**A**) Representative H&E-stained histological sections of wound areas in different groups at day 7 and 14; (**B**) quantitative analysis of the epithelial thickness derived from the H&E staining results; (**C**) Masson’s trichrome-stained sections showing collagen deposition at day 7 and 14; (**D**) quantitative analysis of the collagen deposition area percentage based on Masson’s trichrome staining. The different letters indicate a significant difference among the different groups (*n* = 6 per group, *p* < 0.05).

The microanatomical structures and morphological evolution of the regenerated wound tissues were systematically observed via H&E staining (Figure 7A). After 7 days of treatment, all experimental groups exhibited varying degrees of re-epithelialization; notably, the CMCS/OSA/SAPs composite hydrogel group developed a more continuous and thickened epithelial layer, whereas the blank control group showed only minimal epithelial regeneration. By day 14, a complete neonatal epidermis had formed in all experimental groups, with the composite hydrogel group displaying a more compact epidermal architecture. Quantitative assessment of the dermal thickness at day 7 (Figure 7B) revealed that the blank control group possessed the thinnest dermal layer. In contrast, the dermal thickness in all treatment groups was significantly greater than that of the control. Specifically, the dermal thickness of the composite hydrogel group reached 128.93 μm, showing a statistically significant difference compared to both the blank and pure CMCS/OSA hydrogel groups (*p* < 0.05). Collectively, these findings demonstrate that the CMCS/OSA/SAPs composite hydrogel effectively accelerates the re-epithelialization process and facilitates the rapid structural remodeling and maturation of cutaneous tissues.

#### 2.6.2. Masson’s Trichrome Staining

Collagen plays a pivotal role in the wound healing process by promoting cellular metabolism and providing structural scaffolding for tissue repair. In this study, Masson’s trichrome staining was employed to visualize collagen fibers and evaluate the deposition and organization of collagen within the regenerated tissues (Figure 7C).

The results of Masson’s staining revealed that at day 7, the blank control group exhibited the smallest blue-stained area, indicating relatively low collagen content. By day 14, although a slight increase in collagen deposition was observed in the blank group, it remained markedly lower than that of the experimental groups, highlighting the limited inherent self-healing capacity of skin without intervention. In contrast, the CMCS/OSA/SAPs composite hydrogel group displayed a significantly larger blue-stained area compared to all other groups, characterized by the most dense and organized collagen deposition. Quantitative analysis of the collagen deposition rate at day 7 is presented in Figure 7D. The results indicated that the blank group had the lowest collagen deposition rate (17.33%), whereas all treatment groups showed substantial improvements. Notably, the collagen deposition in the composite hydrogel group reached 45.19%, representing a highly significant difference compared to the blank group (*p* < 0.001). Collectively, the findings of Masson’s staining substantiate that the CMCS/OSA/SAPs composite hydrogel effectively facilitates collagen accumulation, thereby significantly accelerating the overall wound healing process.

#### 2.6.3. CD31 Immunohistochemical Staining

**Figure 8:**
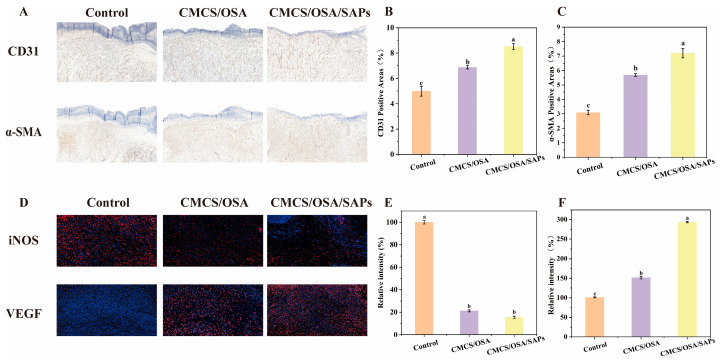
Immunomorphological evaluation of angiogenesis and inflammatory response. (**A**) Representative immunohistochemical (IHC) staining images of CD31 and α-SMA in the wound sections; (**B**,**C**) quantitative analysis of (**B**) CD31-positive areas (%) and (**C**) α-SMA-positive areas based on IHC results; (**D**) immunofluorescence (IF) images of iNOS and VEGF in the wound areas; (**E**,**F**) quantitative analysis of the relative fluorescence intensity for (**E**) iNOS and (**F**) VEGF. The different letters indicate a significant difference among the different groups (*n* = 6 per group, *p* < 0.05).

Neovascularization is a critical determinant of granulation tissue maturation and wound healing, as it provides essential nutrients and oxygen to ensure an adequate blood supply for cellular proliferation, tissue regeneration, and metabolic waste clearance. In this study, IHC staining for platelet endothelial cell adhesion molecule-1 (CD31) was performed to evaluate the angiogenic capacity within the wound tissues (Figure 8A).

The IHC results revealed that the blank control group exhibited sparse brown-yellow positive areas with small capillary diameters. While the CMCS/OSA group showed a discernible trend toward promoting angiogenesis, the CMCS/OSA/SAPs composite hydrogel group displayed the most dense and extensive positive staining, indicating the formation of a robust and intricate vascular network within the regenerated tissue. Quantitative analysis using ImageJ software (V1.54t) (Figure 8B) showed that the percentage of CD31-positive area in the CMCS/OSA/SAPs group was 8.52%, significantly higher than that in the blank control and other experimental groups (*p* < 0.05). This in vivo finding is in high alignment with the trends observed in the previous in vitro HUVEC tube formation assay, confirming that the composite hydrogel, enriched with SAPs, effectively stimulates neovascularization at the wound site.

#### 2.6.4. α-SMA Immunohistochemical Staining

Alpha-smooth muscle actin (α-SMA) serves as a specific marker for smooth muscle cells, and its expression level directly reflects the structural integrity of nascent blood vessels and the extent of wound contraction. In this study, immunohistochemical staining of α-SMA was performed to evaluate the abundance of myofibroblasts and the maturation status of the vasculature within the wound tissues (Figure 8A).

The IHC results revealed varying degrees of α-SMA expression across all groups. In the blank control group, α-SMA signals were weak and sparsely distributed. In contrast, distinct yellowish-brown tubular structures were observed in the CMCS/OSA/SAPs composite hydrogel group, indicating the formation of more mature and functional blood vessels within the wound tissue.

Quantitative analysis using ImageJ (Figure 8C) demonstrated that the CMCS/OSA/SAPs group exhibited the highest α-SMA expression level at 7.2%, which was significantly higher than that of the other groups (*p* < 0.05), while the blank control group showed the lowest expression at 3.1%. These findings suggest that the CMCS/OSA/SAPs composite hydrogel significantly increases the myofibroblast content and promotes vascular maturation, thereby facilitating rapid wound repair and tissue remodeling.

#### 2.6.5. iNOS Immunofluorescence Staining

Inducible nitric oxide synthase (iNOS) is a prototypical marker for M1-type macrophages, and its expression level is closely associated with the severity of tissue inflammation. To further evaluate the inflammatory status of the wound microenvironment during the healing process, immunofluorescence (IF) staining for iNOS was performed on the regenerated tissues (Figure 8D).

The IF results revealed that at day 7 of treatment, the blank control group exhibited intense red fluorescence signals, indicating a high level of local inflammatory response. In contrast, varying degrees of reduced fluorescence intensity were observed in all treatment groups. Notably, the CMCS/OSA/SAPs composite hydrogel group displayed the weakest red fluorescence, demonstrating that the hydrogel effectively downregulates pro-inflammatory factors and mitigates inflammation within the wound microenvironment. Quantitative analysis (Figure 8E) further confirmed that the iNOS expression levels in both the CMCS/OSA and CMCS/OSA/SAPs composite hydrogel groups were significantly lower than those in the blank control group (*p* < 0.05). Collectively, these findings suggest that the CMCS/OSA/SAPs composite hydrogel facilitates a more favorable microenvironment for wound repair by effectively modulating the inflammatory response.

#### 2.6.6. VEGF Immunofluorescence Staining

Vascular endothelial growth factor (VEGF) plays a pivotal role in driving the proliferation of vascular endothelial cells and modulating angiogenic signaling transduction. To further evaluate microvascular regeneration during the healing process, immunofluorescence staining for VEGF was performed on the wound tissues (Figure 8D).

The IF results (Figure 8D) revealed that the blank control group exhibited minimal red fluorescence signals, indicating limited endogenous angiogenic capacity. In contrast, varying degrees of fluorescence enhancement were observed across all treatment groups, with the CMCS/OSA/SAPs composite hydrogel group displaying the most intense and widespread red fluorescence. This observation suggests that the wound tissue in the composite group was in a highly active pro-angiogenic state, favorable for rapid neovascularization. Quantitative analysis (Figure 8F) demonstrated that at day 7 of treatment, the relative VEGF fluorescence intensity in the composite hydrogel group reached 375.2%, which was significantly higher than that of both the blank control and the CMCS/OSA hydrogel groups (*p* < 0.05). These findings robustly confirm that the CMCS/OSA/SAPs composite hydrogel promotes angiogenesis and accelerates the reconstruction of vascular structures by upregulating VEGF expression at the wound site.

### 2.7. Mechanistic Insights into Wound Healing Promotion

The rationale for prioritizing antioxidant capability as a primary indicator lies in the critical role of oxidative stress during the early stages of wound healing. In deep full-thickness skin defects, excessive accumulation of ROS can damage surrounding cells and extracellular matrix components, prolong inflammation, and impair tissue regeneration. Therefore, reducing oxidative stress through the hydrogel system may help establish a favorable microenvironment for wound repair.

Beyond antioxidant activity, SAPs also demonstrated multiple bioactive functions associated with tissue regeneration. In vitro experiments showed that SAPs promoted HUVEC proliferation, migration, and angiogenesis, indicating their positive effects on endothelial regeneration and neovascularization. Consistently, the in vivo results further confirmed enhanced wound healing performance, as evidenced by improved granulation tissue formation, collagen deposition, and vascular regeneration in the CMCS/OSA/SAPs-treated group. In addition, immunohistochemical staining revealed elevated VEGF, CD31, and α-SMA expression, together with reduced iNOS expression, suggesting that the hydrogel may facilitate angiogenesis while alleviating excessive inflammatory responses.

Collectively, these findings indicate that the CMCS/OSA/SAPs hydrogel promotes wound healing through a synergistic mechanism involving antioxidant regulation, inflammation modulation, angiogenesis promotion, and tissue remodeling, demonstrating its potential for functional wound repair applications.

## 3. Conclusions

In this study, oxidized OSA was synthesized via the sodium periodate oxidation method and subsequently cross-linked with the amino groups of CMCS through a Schiff base reaction. This process successfully led to the construction of a dynamic covalent bond hydrogel featuring a sophisticated three-dimensional network. Structural characterizations using FTIR and 1H-NMR confirmed the formation of cross-linked structures. SEM analysis revealed a uniform interconnected porous morphology, which effectively mimics the physical microenvironment of the ECM, providing essential structural support for cell adhesion, migration, and proliferation. Rheological evaluations demonstrated that the hydrogel possesses superior solid-like elastic characteristics and significant self-healing capabilities, enabling rapid structural recovery following mechanical damage.

The therapeutic efficacy of the SAPs-loaded composite hydrogel was comprehensively evaluated using both in vitro cellular models and in vivo animal models. At the cellular level, CCK-8 and live/dead staining assays confirmed the excellent biocompatibility of the hydrogel, with cell viability consistently exceeding 90%. Scratch assays indicated that SAPs significantly induced the horizontal migration of HUVECs. Furthermore, the composite hydrogel markedly enhanced angiogenesis, as evidenced by the increased total tube length and branching points in HUVECs. In a full-thickness skin defect model in SD rats, the composite hydrogel significantly accelerated wound closure, achieving a healing rate of 96% by day 14. Histological analyses, H&E and Masson’s trichrome staining, showed accelerated re-epithelialization and organized collagen deposition. Quantitative analysis verified that the CMCS/OSA/SAPs group significantly shortened the healing cycle compared to the control group. Additionally, immunohistochemical and immunofluorescence results revealed elevated expressions of CD31 (8.52%), α-SMA (7.2%), and VEGF, while lower iNOS levels confirmed the hydrogel’s synergistic anti-inflammatory and regenerative effects.

Despite these promising results, certain limitations remain to be addressed. Future research should focus on the precise identification of bioactive components through mass spectrometry to clarify the specific amino acid sequences and functional peptide fragments of SAPs. Furthermore, multi-omics technologies, such as transcriptomics and proteomics, are needed to elucidate the underlying molecular mechanisms and signaling pathways regulated by SAPs during tissue repair. Although the present study demonstrated the beneficial effects of SAPs on angiogenesis, inflammation regulation, and tissue regeneration, the precise molecular signaling pathways underlying these bioactivities remain unclear. Future studies integrating transcriptomic, proteomic, and pathway-specific analyses are required to further elucidate the upstream regulatory mechanisms and intracellular signaling networks involved in SAPs-mediated wound repair. Finally, the incorporation of additional functional dynamic covalent bonds will be explored to optimize the responsive release kinetics of bioactive peptides, aiming for long-term and precision delivery in complex wound environments.

## 4. Materials and Methods

### 4.1. Materials

In this study, scallop active peptides were obtained from relevant research within our laboratory. SA and CMCS were purchased from Aladdin Bio-Chem Technology Co., Ltd. (Shanghai, China). The HUVEC cells used in this study were purchased from the Cell Resource Center of Shanghai Institute of Biological Sciences, Chinese Academy of Sciences (Shanghai, China; Cat. No. SCSP-5535, Identifier: CSTR:19375.09.3101HUMSCSP5535). All other chemical reagents were of analytical grade.

### 4.2. Preparation of OSA

OSA was prepared via the oxidation of -OH groups in SA using sodium periodate as an oxidant. Specifically, 10 g sodium alginate was dispersed in 50 mL anhydrous ethanol. Subsequently, sodium periodate was dissolved in 100 mL of deionized water and then added to the SA dispersion. The reaction mixture was stirred at 25 °C for 6 h in the dark, followed by the addition of 5 mL ethylene glycol to terminate the reaction, with continued stirring for another 2 h to ensure complete quenching. After the reaction, OSA was precipitated from the mixture by adding 500 mL of anhydrous ethanol. The resulting solid was collected and redissolved in deionized water. The sample was then dialyzed (MWCO: 3500 Da) for 3 d to remove impurities and residual reagents. Finally, the sample was lyophilized to obtain white flocculent OSA powder.

#### FT-IR and 1H-NMR Analysis of OSA

The microstructures of the CMCS/OSA and CMCS/OSA/SAPs hydrogels were examined via scanning electron microscopy (SEM; Quanta 250, Thermo Fisher Scientific, Waltham, MA, USA). Prior to imaging, the hydrogel samples underwent lyophilization, after which thin cross-sections were prepared. These sections were subsequently mounted onto aluminum stubs and sputter-coated with a gold layer to optimize electrical conductivity. Microscopic observations were performed at an accelerating voltage of 10 kV with a magnification of 150×.

### 4.3. Preparation of Hydrogel

6% (*w*/*v*) CMCS solution and 8% (*w*/*v*) OSA precursor solution were prepared using PBS as the solvent. The CMCS/OSA hydrogel was prepared by adding 6% CMCS solution into 8% OSA solution (1:1, *v*/*v*) at room temperature (25 °C) with continuous stirring until the polymers were completely dispersed to form a homogeneous hydrogel. To prepare the CMCS/OSA/SAPs composite hydrogel, an appropriate amount of SAPs was incorporated into the CMCS/OSA hydrogel precursor solution under continuous stirring, followed by mixing CMCS and OSA in equal proportions until a uniform system was obtained. The resulting hydrogel was then subjected to vacuum degassing to remove bubbles and stored at 4 °C until further use.

The choice of polymer concentrations was determined through preliminary screening of gelation kinetics and operability. Lower polymer concentrations resulted in excessively prolonged gelation times and mechanically weak frameworks. Conversely, higher concentrations led to rapid localized cross-linking, causing immediate clogging and poor network homogeneity. The 6%/8% ratio was therefore selected to achieve a balanced dynamic network with a controlled cross-linking velocity, which ensures appropriate handling properties for practical application.

### 4.4. Characterization of Hydrogel

#### 4.4.1. Macroscopic and Microscopic Morphological Analysis

The sol–gel transition of the mixture was determined by the tube inversion method. Specifically, the mobility of CMCS/OSA and CMCS/OSA/SAPs hydrogels prepared using the above method was evaluated by inverting the vials to observe their flow conditions. In this system, the formed hydrogel characterized by a three-dimensional network could immobilize water molecules, thereby preventing the mixture from flowing when the vial was inverted, which was used to confirm the gel state.

The morphologies of CMCS/OSA and CMCS/OSA/SAPs hydrogels were observed by SEM (Quanta 250, Thermo Fisher Scientific, Waltham, MA, USA). Before SEM, the CMCS/OSA and CMCS/OSA/SAPs hydrogels were freeze-dried and a thin cross-section was cut out, fixed on the aluminum sample stubs, and coated with gold to enhance conductivity for SEM. The morphology of each hydrogel was observed at 10 kV extra-high tension with a magnification of 150×.

#### 4.4.2. Rheological Analysis

The rheological properties of the CMCS/OSA and CMCS/OSA/SAPs hydrogels were analyzed using a rotational rheometer (Anton-Paar MCR 302, Anton-Paar, Graz, Austria) equipped with a PP25 parallel-plate rotor (25 mm diameter) at a constant temperature of 25 °C. After loading, the samples were left to equilibrate for 5 min. For dynamic rheological analysis, a strain sweep (0.1–500%) was first performed at 10 rad/s to determine the LVE region. Subsequently, the G′ and G″ were monitored at a constant strain of 1% with the angular frequency varied from 0.1 to 100 rad/s.

The structural recovery capability of the hydrogels was evaluated by alternating step-strain measurements. At 10 rad/s, shear strains of 1% and 300% were applied alternately. The cycles were repeated 5 times, and the real-time recovery of G′ and G″ was recorded. For macroscopic self-healing observation, the Sudan Red-labeled hydrogel and the unstained sample were cut in half and placed in contact. After standing at room temperature, the self-healing effect was visually evaluated by observing the interfacial fusion and dye diffusion.

#### 4.4.3. Swelling Studies

The swelling behavior of the hydrogels was evaluated using a gravimetric method. The lyophilized hydrogel samples were accurately weighed (W_0_) and then immersed in PBS solution at 37 °C. At predetermined time intervals, the samples were removed, gently blotted with filter paper to remove excess surface liquid, and reweighed (W_t_).

The swelling ratio (SR, %) was calculated according to Equation (1):

(1) $$\mathrm{SR}\left(\%\right)\;=\;\frac{W_{t}-W_{0}}{W_{0}}\times 100\%$$

where W_0_ is the initial weight of the sample and W_t_ is the wet weight at time t.

All experiments were performed in triplicate, and results are expressed as means ± standard deviations (SDs).

### 4.5. Biocompatibility Evaluation

HUVECs were selected as an in vitro cell model to evaluate both the biocompatibility and pro-angiogenic properties of the hydrogels. HUVECs are widely used in wound healing studies because endothelial cell proliferation, migration, and angiogenesis are essential biological events during the proliferative stage of tissue repair, particularly in the reconstruction of vascular networks within damaged tissues. Since insufficient vascularization and impaired angiogenesis are recognized as critical limitations in full-thickness wound healing, HUVEC-based assays were considered highly relevant for evaluating the regenerative potential of the CMCS/OSA/SAPs hydrogel system. Therefore, CCK-8 assay and Live/Dead staining were performed to assess cytocompatibility, while cell migration and tube formation assays were conducted to investigate the potential of the hydrogels to promote endothelial regeneration and angiogenesis.

#### 4.5.1. Hydrogel Pretreatment

The prepared hydrogel dressings were sterilized by exposure to ultraviolet (UV) light (T-200W, Foshan Yichen Electronic Technology Co., Ltd., Foshan, China) for 2 h in a sterile environment. To obtain the hydrogel leaching liquor, these sterilized samples were placed on a microplate and incubated with serum-free culture medium for 24 h. The resulting leaching liquor was then filtered through a 0.22 μm membrane to ensure sterility. The filtered leaching liquor was collected and utilized for subsequent cell experiments. This dual-stage sterilization process, which couples the macro-scale UV irradiation of the dynamic solid matrix with the 0.22 μm membrane filtration of the liquid extract, effectively guarantees a robust sterile microenvironment for cell culturing. While explicit microbial colony counting was not utilized during this platform screening phase, this classical combination of physical irradiation and size-exclusion filtration has been universally established to thoroughly eliminate bacterial and fungal vectors, successfully mitigating potential microbial interference for subsequent in vitro biological evaluations.

#### 4.5.2. In Vitro Cytotoxicity Assay

The in vitro cytocompatibility of the drugs together with the hydrogel leaching liquor was evaluated using a Cell Counting Kit-8 (Beyotime^®^, Beyotime Institute of Biotechnology, Shanghai, China) (CCK-8). Briefly, 96-well culture plates were utilized to harbor the cells at an initial concentration of 8 × 10^4^ cells/mL, allowing them a 24 h window to achieve proper surface adherence. Next, the depleted culture supernatant was completely evacuated, and 100 µL of either drug solution or hydrogel leaching liquor was added for a further incubation of 24~48 h. After treatment, 110 µL of detection solution (containing 100 µL of fresh medium and 10 µL of the colorimetric kit reagent) was introduced into individual wells, followed by a 30–60 min thermal incubation phase. Ultimately, optical density (OD) tracking at a wavelength of 450 nm was executed via a microplate reader (Multiskan FC, Thermo Fisher Scientific, Waltham, MA, USA), and the cell viability of each group was calculated according to Equation (2):

(2) $$\mathrm{Cell}\;\mathrm{viability}\left(\%\right)\;=\;\frac{A_{a}-\;A_{0}}{A_{c}-\;A_{0}}\;\times \;100\%$$

where A_a_ is the absorbance of the wells containing cells, CCK-8 solution, and drug/leaching liquor; A_0_ is the absorbance of the wells containing culture medium and CCK-8 solution without cells; and A_c_ is the absorbance of the wells containing cells and CCK-8 solution without drugs.

#### 4.5.3. Live/Dead Cell Staining

The effects of the drugs together with the hydrogels on cell viability were evaluated using a Calcein-AM/PI Double Staining Kit (Beyotime^®^, Beyotime Institute of Biotechnology, Shanghai, China). In short, cells were seeded into 24-well plates at a density of 1 × 10^5^ cells/mL (500 μL/well) and pre-incubated for 24 h to allow for cell attachment. Subsequently, the original medium was discarded, and 500 μL of either drug solution or hydrogel leaching liquor was added for a further incubation of 24–48 h. After treatment, 300 μL of buffer and 0.3 μL of double staining reagent (at a ratio of 1:1000) were added to each well and incubated in an O_2_ incubator (BB150, Thermo Fisher Scientific, Waltham, MA, USA). for 10 min in the dark. The cells were then observed and photographed using a fluorescence microscope. The cytotoxicity of the samples was evaluated based on the distribution and intensity of the green fluorescence from live cells.

#### 4.5.4. Hemocompatibility Analysis

The hemocompatibility of the samples was evaluated via the erythrocyte hemolysis method. Fresh blood was first obtained from the rat heart and immediately anticoagulated, followed by centrifugation for 3 min. The supernatant was discarded, and the red blood cells (RBCs) were washed three times with physiological saline to remove impurities. Finally, the RBCs were resuspended in physiological saline to prepare a 2% (*v*/*v*) erythrocyte suspension.

For the hemolysis assay, 100 μL of each sample (SAPs solution, CMCS/OSA leaching liquor, and CMCS/OSA/SAPs leaching liquor) was mixed with 1 mL of the erythrocyte suspension. Physiological saline and Triton X-100 were utilized as the negative and positive controls, respectively. After incubation at 37 °C for 2 h, the mixtures were centrifuged again for 3 min to collect the supernatant. The absorbance at 540 nm was measured using a UV-Vis spectrophotometer (Lambda-900, PerkinElmer, Waltham, MA, USA), and the hemolysis rate was calculated according to Equation (3):

(3) $$\mathrm{Hemolysis}\;\mathrm{rate}\;\left(\%\right)\;=\;\frac{{\mathrm{OD}}_{\mathrm{sample}}-\;{\mathrm{OD}}_{\mathrm{NC}}}{{\mathrm{OD}}_{\mathrm{PC}}-\;{\mathrm{OD}}_{\mathrm{NC}}}\;\times \;100\%$$

where OD_sample_ is the absorbance of the experimental group, OD_NC_ is the absorbance of the negative control group, and OD_PC_ is the absorbance of the positive control group.

### 4.6. Evaluation of In Vitro Biological Activities

#### 4.6.1. Cell Migration Assay

HUVECs were seeded into 24-well plates at a density of 2 × 10^5^ cells/well and cultured until 80% confluence was reached. Subsequently, a 200 μL sterile pipette tip (Biosharp Life Sciences, Hefei, China). was utilized to create a distinct scratch across the cell monolayer. The wells were gently rinsed with PBS to remove cell debris, followed by the addition of serum-free hydrogel leaching liquor according to the experimental groups. The plates were then incubated at 37 °C in a 5% CO_2_ atmosphere for 24~48 h. Images were captured at 0, 24, and 48 h post-scratching using an inverted microscope (Eclipse 50i, Nikon, Tokyo, Japan). The migration process was quantitatively analyzed using ImageJ (V1.54t), and the wound migration rates were calculated according to Equation (4):

(4) $$\mathrm{Cell}\;\mathrm{migration}\;\mathrm{rate}\;\left(\%\right)\;=\;\frac{S_{i}-S_{0}}{S_{0}}\times 100$$

where S_0_ is the initial wound area at 0 h, and S_i_ is the remaining wound area at 24 or 48 h.

#### 4.6.2. Angiogenesis Assay

The effect of the samples on the angiogenic ability of HUVECs was evaluated via an in vitro tube formation assay. Prior to the experiment, HUVECs were subjected to starvation by culturing in serum-free medium for 24 h. To ensure a uniform coating, 24-well plates and pipette tips were pre-chilled at −20 °C. Subsequently, 50 μL of Matrigel was added to each well and incubated at 37 °C in a 5% CO_2_ atmosphere for 30 min to allow for gelation. HUVECs were then seeded onto the Matrigel-coated wells at a density of 1 × 10^5^ cells per well in 200 μL of culture medium containing the test samples. After incubation for 6~12 h, the formation of capillary-like structures was observed and captured using an inverted microscope (Eclipse 50i, Nikon, Tokyo, Japan). Quantitative analysis of the tubular structures was performed using the Angiogenesis Analyzer plugin for ImageJ.

#### 4.6.3. Antioxidant Activity Evaluation

To investigate the influence of SAPs concentration on the antioxidant capacity of the hydrogels, CMCS/OSA/SAPs composite hydrogels were fabricated with varying SAPs loading concentrations (2, 4, 6, 8, and 10 mg/mL) according to our previously established methods. The in vitro antioxidant performance of the hydrogel samples was assessed using DPPH^+^·, hydroxyl (·OH), and ABTS^+^· radical scavenging assays.

For the DPPH assay, a 0.1 mM DPPH solution was prepared in anhydrous ethanol and stored protected from light. An appropriate amount of the hydrogel was dispersed in ethanol under constant stirring and then centrifuged to collect the clear supernatant. Subsequently, the sample solution was mixed with the DPPH solution at a specific ratio (1:1, *v*/*v*) and allowed to react in the dark at room temperature for 30 min. The reduction in absorbance at 517 nm was monitored using a microplate reader (Multiskan FC, Thermo Fisher Scientific, Waltham, MA, USA). A DPPH solution without the hydrogel served as the reference, while ascorbic acid (V_c_) was utilized as the positive control. The DPPH radical scavenging rate was calculated using Equation (5):

(5) $${\mathrm{DPPH}}^{+}\cdot \mathrm{scavenging}\;\mathrm{rate}\left(\%\right)=\frac{\left(A_{0}\;-{\;A}_{s}\right)}{A_{0}}\times 100\%$$

where A_0_ is the absorbance of the blank DPPH solution and A_s_ is the absorbance of the sample.

The ·OH scavenging activity was determined as follows: Initially, a salicylic acid solution (9 mmol/L) and a FeSO4 solution (9 mmol/L) were prepared using anhydrous ethanol and deionized water, respectively. Meanwhile, a standard H_2_O_2_ solution was diluted to 8.8 mmol/L for further use. At the beginning of the assay, sample solutions of various concentrations were placed in test tubes, to which 1 mL of FeSO_4_ solution and 1 mL of salicylic acid solution were sequentially added. Finally, 1 mL of H_2_O_2_ solution was introduced to initiate the reaction. The mixtures were allowed to react in the dark for 30 min. Upon completion of the reaction, each experimental group was centrifuged, and the supernatant was collected. The absorbance was measured at 510 nm using a microplate reader (Multiskan FC, Thermo Fisher Scientific, Waltham, MA, USA). Deionized water served as the blank control, while Vc was utilized as the positive control. The ·OH scavenging rate was calculated according to Equation (6):

(6) $$\cdot \mathrm{OH}\;\mathrm{scavenging}\;\mathrm{rate}\left(\%\right)\;=\;\frac{\left(A_{0}-A_{s}\right)}{A_{0}}\;\times \;100\%$$

where A_0_ is the absorbance of the blank OH solution and A_s_ is the absorbance of the sample.

The ABTS^+^• radical scavenging assay was conducted by mixing equal volumes (1:1, *v*/*v*) of 7 mM ABTS and 2.45 mM potassium persulfate solutions. This blend was kept at room temperature in the dark for 16 h to generate the cation radicals, then diluted with PBS to attain an initial absorbance of 0.7 ± 0.02 at 734 nm. Subsequently, 2 mL of this working solution was transferred into tubes containing graded concentrations of samples, reacting for 30 min in a dark incubator. Following a brief centrifugation step to isolate the supernatant, its 734 nm optical density was quantified using a Multiskan FC reader (Thermo Fisher Scientific, USA). The baseline control excluded the hydrogel component, and Vc acted as the positive benchmark. The percentage inhibition was deduced via Equation (7):

(7) $${\mathrm{ABTS}}^{+}\cdot \mathrm{scavenging}\;\mathrm{rate}\left(\%\right)\;=\;\frac{\left(A_{0}-A_{s}\right)}{A_{0}}\;\times \;100\%$$

where A_0_ is the absorbance of the blank ABTS solution and A_s_ is the absorbance of the sample.

### 4.7. Animal Experiment Design

This study utilized 6–8-week-old male SPF SD rats (weighing 250–300 g) purchased from Fuzhou Wushi Experimental Animal Trading Co., Ltd. (Fuzhou, China). These rodents were maintained within an SPF barrier facility under a controlled diurnal rhythm (12 h light/dark cycle) and a relative humidity of 50–70%, with free access to clean water and standard laboratory chow. The institutional animal care guidelines were strictly vetted and authorized by the Fuzhou University Animal Care and Use Committee under authorization number 2023-SG-049, conforming strictly to the European Community directive (2010/63/EU) concerning laboratory animal welfare.

Before initiation of the trial, a 7-day stabilization period was provided for the rats to adapt to their new environment, with a stocking density of three individuals per cage. Following this, random allocation divided the 18 subjects into three distinct operational branches (*n* = 6 per group). Defects in the blank reference cohort involved only the full-thickness cutaneous removal without receiving any secondary therapeutics, thereby representing a negative control. For the active therapeutic cohorts (*n* = 6), full-thickness dorsal skin defects were systematically established. Post-surgery, the respective groups were topically dressed with either CMCS/OSA hydrogels or composite CMCS/OSA/SAPs dressings. These protocols aimed to assess and contrast the structural efficiency of both hydrogel matrices in accelerating tissue repair.

In detail, general anesthesia was successfully achieved using volatilized isoflurane inhalation, after which the dorsal fur was clipped and sanitized using a 75% ethanol solution. A round, full-thickness integumentary wound measuring 10 mm in diameter was stamped at the dorsal midline via a sterile punch tool. The cutting process penetrated entirely through the dermal structures down to the loose subcutaneous layers, while safeguarding the underlying muscular framework. To eliminate variation regarding wound dimensions and operational depth, all surgical steps were carried out by one individual investigator employing identical instruments.

### 4.8. Animal Sample Collection and Determination

Prior to tissue harvesting, the rats were fasted for 12 h. The skin tissues surrounding the wound site were collected and fixed in a 10% neutral buffered formalin solution, followed by dehydration and embedding in paraffin wax. Subsequently, the paraffin-embedded tissues were sectioned into slices. H&E staining and Masson’s trichrome staining were performed to evaluate the tissue morphology and collagen deposition, respectively.

Furthermore, IHC staining was employed to detect the expression of CD31 and α-SMA. For the qualitative and quantitative analysis of VEGF and iNOS, IF techniques were utilized. All stained sections were observed and captured using an inverted microscope (Eclipse 50i, Nikon, Tokyo, Japan).

### 4.9. Statistical Analysis

All experimental measurements were performed at least in triplicate, and each group in the in vivo wound healing assay consisted of six animals (*n* = 6). Data are expressed as means ± standard deviations (SDs). Statistical analysis was performed using SPSS 22.0 software. The normality of the data distribution was verified using the Shapiro–Wilk test before parametric analyses. Differences among multiple groups were evaluated using one-way ANOVA followed by Duncan’s multiple range test, whereas Student’s t-test was employed for two-group statistical comparisons. A value of *p* < 0.05 was considered statistically significant.

## Data Availability

The original contributions presented in this study are included in the article. Further inquiries can be directed to the corresponding author.
